# Geometrical and functional changes of left heart in adults with prehypertension and hypertension: a cross-sectional study from China

**DOI:** 10.1186/s12872-016-0286-3

**Published:** 2016-05-28

**Authors:** Tan Li, Jun Yang, Xiaofan Guo, Shuang Chen, Yingxian Sun

**Affiliations:** Department of Cardiovascular Ultrasound, The First Affiliated Hospital of China Medical University, Shenyang, People’s Republic of China; Department of Cardiology, The First Affiliated Hospital of China Medical University, 155 Nanjing North Street, Heping District, Shenyang, 110001 People’s Republic of China

**Keywords:** Echocardiography, Hypertension, Prehypertension, Left ventricular geometry

## Abstract

**Background:**

Studies regarding the association between prehypertension and the structual changes of left heart are scanty. However, which type of the geometrical change of left heart is predominated one in prehypertension and hypertension is controversial. It is therefore important to investigate geometrical and functional changes of left heart in adults with prehypertension and hypertension because of their prognostic significance.

**Methods:**

The study was based on a cross-sectional design, a total of 10547 participants were classified into normotension group, prehypertension group and hypertension group. We analyzed clinical characteristics, echocardiographic parameters and distribution of left ventricular (LV) geometrical patterns in different groups.

**Results:**

Participants with prehypertension had higher values of most of echocardiographic parameters than those with normotension. The prevalence of left ventricular hypertrophy(LVH) was statistically different among three groups (*P* <0.001), and the rates of LVH in the three groups were 5.9, 8.6, 28.4 % by indexation to height^2.7^ and 4.9, 5.3, 19.3 % by indexation to BSA, respectively. The prevalence rates of eccentric hypertrophy, concentric remodeling and concentric hypertrophy were 7.3 %, 5.3 % and 1.4 % in prehypertension group, and 17.8 %, 8.8 % and 10.6 % in hypertension group. Logistic regression analysis showed that systolic blood pressure (SBP), diastolic blood pressure (DBP) and mean artery pressure (MAP) were all independent risk factors for left cardiac structural changes, and pulse pressure (PP) was independent risk factor for concentric and eccentric hypertrophy. Among four indices, DBP levels with OR values of 1.192, 1.759 and 1.278 were the strongest indicator for concentric remodeling, concentric hypertrophy and eccentric hypertrophy, respectively (*P* <0.001).

**Conclusions:**

There exists LV geometrical change in adults with prehypertension and hypertension in rural Chinese population, and the eccentric hypertrophy was the highest proportion of geometric alterations. SBP, DBP, MAP and PP were all positively associated with left cardiac structural changes, and the association of DBP was the strongest.

## Background

The type of LV geometrical change is determined mainly by the predominating type of stimulus to the myocardium, volume overload or pressure overload in hypertension [1]. Systolic and diastolic function of the left ventricle can be significantly influenced by the geometrical patterns which are also important in cardiovascular prognosis [2]. Three types of the geometrical change of the left ventricle include concentric remodeling, concentric hypertrophy, and eccentric hypertrophy [3]. The geometrical patterns vary from one patient to another, however, which is predominated type in hypertension and prehypertension is controversial. Prehypertension, as a transition from the normal blood pressure (BP) to the diagnosis of hypertension, is considered as a starting point in the cardiovascular disease (CVD) [4], has gradually attracted more attention in recent years. In 2003, the United States JNC7 defined prehypertension as SBP of 120–139 mmHg and (or) DBP of 80–89 mmHg [5]. Although persons do not have symptoms in prehypertensive stage, some geometrical and functional changes of the heart may be detected by echocardiography. Some investigators had reported that hypertensive patients had higher prevalence of geometrical and functional changes in left heart [6–8], but data regarding the association between prehypertension and geometrical and functional changes of left heart are scanty, especially in rural adults from Northeast China, where hypertension is prevalent. Therefore, we conducted this study to investigate geometrical and functional changes of left heart in adults with prehypertension and hypertension in Northeast rural Chinese population.

## Methods

### Study population

Liaoning Province is located in Northeast China. From January 2012 to August 2013, a representative sample aged ≥ 35 years was selected to describe the prevalence, incidence and natural history of cardiovascular risk factors in rural areas of Liaoning Province. The study adopted a multi-stage, stratified randomly cluster-sampling scheme. In the first stage, 3 counties (Dawa, Zhangwu, and Liaoyang County) were selected from the eastern, southern, and Northern region of Liaoning province. In the second stage, one town was randomly selected from each county (a total of 3 towns). In the third stage, 8–10 rural villages from each town were randomly selected (a total of 26 rural villages). Participants with pregnancy, malignant tumor and mental disorder were excluded. All the eligible permanent residents aged ≥ 35 years from each village were invited to attend the study (a total of 14016 participants). Of those, 11956 participants agreed and completed the present study and the response rate was 85.3 %. The study was approved by the Ethics Committee of China Medical University (Shenyang, China). All procedures were performed in accordance with the ethical standards. Written consent was obtained in all participants after they had been informed of the objectives, benefits, medical items and confidentiality agreement of personal information. If the participants were illiterate, we obtained the written informed consents from their proxies. In this report, subjects were excluded if they met any of the following criteria: congenital heart disease, cardiac tumor, myocardial infarction, pericardia disease, cardiomyopathy or other organic heart disease, all kinds of serious arrhythmia, heart valve disease (the valve with moderate to severe reflux or narrow). Finally, a total of 10547 participants were available for analysis, 3616 subjects with prehypertension, 5280 patients with hypertension and 1651 participants with normotension.

### Blood pressure measurement

According to American Heart Association protocol, BP was measured three times at 2 min intervals after at least 5 min of rest using a standardized automatic electronic sphygmomanometer (HEM-907; Omron), which had already been validated according to the British Hypertension Society protocol [9]. The participants were advised to avoid caffeinated beverages and exercise for at least 30 min before the measurement. During the measurement, participants were seated with the arm supported at the level of the heart. The mean of three BP measures were averaged for analysis. According to the United States JNC7, PH was defined as SBP of 120–139 mmHg and (or) DBP of 80–89 mmHg. Hypertension was diagnosed as a SBP ≥140 mmHg and (or) a DBP ≥90 mmHg. Normotensive controls had SBP <120 mmHg and DBP <80 mmHg. PP was calculated by (SBP-DBP) and MAP was calculated by 1/3(SBP + 2DBP).

### Echocardiography measurements

The echocardiograms were obtained using a commercially available Doppler echocardiograph (Vivid, GE Healthcare, United States), with a 3.0-MHz transducer. The transthoracic echocardiogram included M-mode, 2-dimensional, spectral and color Doppler with subjects in the supine position. Echocardiogram analyses and readings were performed by three physicians specialized in echocardiography. The parasternal acoustic window was used to record two-dimensional and M-mode images of the LV internal diameter, wall thickness, aortic root and left atrium. The apical acoustic window was used to record 4- and 5-chamber images.

Correct orientation of planes for imaging and Doppler recordings was verified using previously described procedures [10]. All M-mode tracings were obtained at 50 mm/s. LV end-diastolic internal dimension (LVIDd), LV end-systolic internal dimension (LVIDs), interventricular septal thickness (IVSd), posterior wall thickness (PWTd) and left atrial diameter (LAD), as well as aortic annular diameter (AOD) were performed according to the guidelines of the American Society of Echocardiography and the European Association of Echocardiography [11, 12].

Left ventricular mass (LVM) was estimated by Devereux’ s formula according to the American Society of Echocardiography simplified cubed equation [13]. LVM = 0.8 × [1.04{(IVSTd + PWTd + LVIDd)^3^–LVIDd^3^}] + 0.6 g.

LVM was divided by height^2.7^ and body surface area (BSA) to obtain left ventricular mass index (LVMI_ht2.7_ and LVMI_BSA_). LVH was diagnosed using the following defining criteria [14, 15]: >48 g/m^2.7^ and 44 g/m^2.7^ for men and women respectively when LVM was indexed to height^2.7^, and >115 g/ m^2^ and >95 g/ m^2^ for men and women respectively when LVM was indexed to BSA. The relative wall thickness (RWT) was calculated using the formula (IVSd + PWTd) / LVIDd. Increased RWT was present when this ratio was >0.42 [16].

LV geometry was classified into four patterns using LVMI_ht2.7_ and RWT values [8, 14]. These are normal geometry when LVMI was normal and RWT ≤ 0.42, concentric remodeling when LVMI was normal and RWT > 0.42, concentric hypertophy when LVMI was increased and RWT > 0.42, eccentric hypertrophy when LVMI was increased and RWT ≤ 0.42. The LV end-diastolic volume(LVEDV) and LV end-systolic volume(LVESV) were determined using the Teichholz equations [17]: LVEDV(ml) = LVIDd^3^ × 7.0/(2.4 + LVIDd), LVESV(ml) = LVIDs^3^ × 7.0/(2.4 + LVIDs). Using LVEDV and LVESV, the LV ejection fraction (EF) was calculated as [(LVEDV-LVESV)/LVEDV] × 100 % and LV fractional shortening (FS) was calculated as [(LVIDd-LVIDs)/LVIDd] × 100 %. All Doppler echocardiographic recordings were registered with 100 mm/s and performed in expiration. Examinations of mitral inflow were performed by pulsed wave Doppler with the sample volume at the mitral valve leaflet tips in the apical four-chamber view and early transmitral diastolic peak flow (E) and atrial peak flow (A) and E/A ratio were determined [16]. Diastolic dysfunction was defined as E/A <1. The individuals who presented pseudonormalization, defined as E/A at least one in conjunction with end-systolic LAD, in parasternal long axis view, more than 3.8 cm for women and 4.0 cm for men, were also considered as having diastolic dysfunction [18].

### Covariate measurements and definitions

Information on covariates, such as age, gender and lifestyle, were collected during a single clinic visit by cardiologists and trained nurses using a standard questionnaire by face-to-face interview. Before the survey was performed, we invited all eligible investigators to attend the organized training. The training contents included the purpose of this study, how to administer the questionnaire, the standard method of measurement, the importance of standardization, and the study procedures. A strict test was evaluated after this training, only those who scored perfectly on the test could become investigators. During data collection, our inspectors had further instructions and support.

Weight and height were measured to the nearest 0.1 kg and 0.1 cm respectively with the participants in light weight clothing and without shoes. Waist circumference (WC) was measured at the umbilicus using a non-elastic tape (to the nearest 0.1 cm), with the participants standing at the end of normal expiration. Body mass index (BMI) was calculated as weight in kilograms divided by the square of the height in meters. BSA was calculated as [0.0061 × height (cm) + 0.0128 × weight (kg)-0.1529].

Fasting blood samples were collected in the morning after at least 12 h of fasting for all participants. Blood samples were obtained from an antecubital vein into Vacutainer tubes containing EDTA. Fasting plasma glucose (FPG), total cholesterol (TC), low-density lipoprotein cholesterol (LDL-C), high-density lipoprotein cholesterol (HDL-C), triglyceride (TG), plasma creatine (PC), uric acid (UA) and other routine blood biochemical indexes were analyzed enzymatically on an autoanalyzer. All laboratory equipment was calibrated and blinded duplicate samples were used.

### Statistical analysis

Continuous variables were presented as the means and standard deviation (SD), and categorical variables were expressed as numbers or percentages. Differences among categories were evaluated using ANOVA, non-parametric test or the *χ*2-test as appropriate. Multivariate logistic regression analyses were used to identify independent factors of SBP, DBP, MAP, and PP with odds ratios (ORs) and corresponding 95 % confidence intervals (CIs) calculated. All the statistical analyses were performed using SPSS version 17.0 software, and P values less than 0.05 were considered statistically significant.

## Results

### Clinical characteristics

As shown in Table 1, of the 10547 participants, 54.2 % participants were women, 5280 (50.1 %) and 3616 (34.3 %) met JNC-7 criteria for prehypertension and hypertension, respectively. Compared with the normotension group, hypertension and prehypertension groups were more likely to have higher weight, WC, BSA and BMI. Both the hypertension and prehypertension groups had higher SBP, DBP, MAP, PP, TG, TC, LDL-C, FPG, hemoglobin and UA than the normotension group. The hypertension group had significantly higher PC, but it did not differ statistically between prehypertension and normotension group.

**Table 1:** Baseline characteristics for normotension, prehypertension and hypertension

Variables	NT	PH	HT
	*N* = 1651	*N* = 3616	*N* = 5280
Age	48.4 ± 9.0	50.9 ± 9.7*	57.1 ± 10.1#†
Males, n (%)	544 (32.9)	1728 (47.8)	2553 (48.4)
Han ethnicity, n (%)	1575	3428	5014
Height, cm	160.1 ± 7.5	161.3 ± 8.2*	160.2 ± 8.4#
Weight, kg	59.5 ± 9.6	63.6 ± 11.1*	65.8 ± 11.5#†
WC, cm	77.7 ± 8.6	80.9 ± 9.3*	84.8 ± 9.7#†
BSA, m2	1.59 ± 0.15	1.64 ± 0.18*	1.67 ± 0.18#†
BMI, kg/ m^2^	23.2 ± 3.3	24.4 ± 3.5*	25.6 ± 3.6#†
BMI ≥ 30 kg/ m^2^, n (%)	2.4	5.9	10.5**
Current smoke, n (%)	32.6	36.6	34.7**
Current drinking, n (%)	12.6	23.8	24.5**
Heart rate, bmp	76 ± 11	77 ± 12*	79 ± 14#†
SBP, mmHg	112.1 ± 5.8	129.2 ± 5.8*	158.7 ± 19.3#†
DBP, mmHg	69.1 ± 5.8	77.7 ± 6.4*	88.8 ± 11.0#†
MAP, mmHg	83.5 ± 5.1	94.8 ± 5.1*	112.1 ± 11.7#†
PP, mmHg	43.0 ± 5.8	51.6 ± 7.4*	69.9 ± 17.4#†
TC, mmol/L	4.9 ± 1.0	5.1 ± 1.0*	5.4 ± 1.1#†
TG, mmol/L	1.3 ± 1.1	1.5 ± 1.3*	1.8 ± 1.6#†
LDL-C, mmol/L	2.6 ± 0.7	2.8 ± 0.8*	3.1 ± 0.9#†
HDL-C, mmol/L	1.4 ± 0.3	1.4 ± 0.4*	1.4 ± 0.4†
FPG, mmol/L	5.5 ± 1.2	5.7 ± 1.3*	6.2 ± 1.9#†
PC, mg/dl	70.1 ± 11.6	71.2 ± 12.8	72.9 ± 27.3#†
Hemoglobin, g/dl	132.8 ± 17.2	138.6 ± 20.0*	140.6 ± 18.1#†
UA, umol/L	270.7 ± 77.2	285.2 ± 81.3*	301.0 ± 87.2#†
Antihypertensive medication, n (%)	0	0	1525 (28.9)

NT indicates normotension; PH, prehypertension; HT, hypertension

*P < 0.001 between PH and NT, #*P* < 0.001 between HT and PH, †*P* < 0.001 between HT and NT, ** *P* < 0.001 among three groups

### Echocardiographic parameters comparison

Compared with the normotension group, LVIDd, LVIDs, LVM, LVMI, AOD, LAD, LAD/BSA, A wave, LVEDV, LVESV and SV were significantly higher, while E wave and E/A were significantly lower in prehypertension group (*P* <0.05). Among the three subgroups, hypertension group had the highest IVSd, PWTd, LVIDd, LVIDs, LVM, LVMI, RWT, AOD, LAD, LAD/BSA, A wave, LVEDV, LVESV and SV, and the lowest E wave, E/A, LVEF and FS (*P* <0.001). The prevalence of LVH was statistically different among three groups (*P* < 0.001), and the rates of LVH in the three groups were 5.9, 8.6, 28.4 % by indexation to height^2.7^ and 4.9, 5.3, 19.3 % by indexation to BSA, respectively (Table 2).

**Table 2:** Echocardiographic parameters comparison of normotension, prehypertension and hypertension

Variables	NT	PH	HT
	*N* = 1651	*N* = 3616	*N* = 5280
IVSd, cm	0.83 ± 0.18	0.84 ± 0.12	0.93 ± 0.29#†
LVIDd, cm	4.56 ± 0.37	4.68 ± 0.40*	4.76 ± 0.43#†
LVIDs, cm	3.0 ± 0.37	3.08 ± 0.40&	3.15 ± 0.43#†
PWTd, cm	0.83 ± 0.22	0.84 ± 0.30	0.89 ± 0.22#†
LVM, g	122.7 ± 65.8	133.6 ± 74.5*	153.1 ± 103#†
LVMI_ht2.7_, g/m^2.7^	34.5 ± 19.1	36.8 ± 20.7*	42.9 ± 27.7#†
LVMI_BSA,_ g/m^2^	77.4 ± 41.2	81.3 ± 44.9*	91.7 ± 59.5#†
RWT	0.36 ± 0.07	0.36 ± 0.08	0.38 ± 0.09#†
LVH, height^2.7^, n (%)	97 (5.9)	272 (8.6)	1500 (28.4)**
LVH, BSA, n (%)	81 (4.9)	192 (5.3)	1019 (19.3)**
AOD, cm	2.16 ± 0.25	2.22 ± 0.31*	2.28 ± 0.30#†
LAD, cm	3.20 ± 0.35	3.31 ± 0.37*	3.45 ± 0.40#†
LAD/BSA, cm/ m^2^	2.03 ± 0.23	2.03 ± 0.24*	2.08 ± 0.26#†
E wave, cm/s	76.6 ± 19.0	75.0 ± 19.4&	70.1 ± 18.8#†
A wave, cm/s	67.5 ± 15.2	72.0 ± 15.8*	81.2 ± 17.7#†
E/A	1.19 ± 0.38	1.09 ± 0.37*	0.91 ± 0.34#†
LVEDV, ml	96.3 ± 18.3	102.5 ± 21.0*	106.6 ± 22.8#†
LVESV, ml	35.7 ± 10.7	38.4 ± 12.3&	40.7 ± 13.6#†
SV, ml	60.6 ± 14.8	64.1 ± 17.8&	65.9 ± 19.2#†
LVEF, %	62.82 ± 8.88	62.41 ± 9.87	61.67 ± 10.33#†
FS, %	34.27 ± 6.46	34.14 ± 7.13	33.68 ± 7.39#†

*P < 0.001 between PH and NT, &*P* < 0.05 between PH and NT, #*P* < 0.001 between HT and PH, †*P* < 0.001 between HT and NT, ** *P* < 0.001 among three groups

### Distribution of LV geometrical pattern

Classification of LV geometry showed that the eccentric hypertrophy was the highest proportion of geometric alterations in hypertension and prehypertension groups.in the present study population. The prevalence rates of eccentric hypertrophy, concentric remodeling and concentric hypertrophy were 7.3 %, 5.3 % and 1.4 % in prehypertension group, and 17.8 %, 8.8 % and 10.6 % in hypertension group (Table 3).

**Table 3:** Classification of LV geometry according to gender and BP groups

Classification	NT	PH	HT	*P*
	(*N* = 1651)	(*N* = 3616)	(*N* = 5280)	
Normal geometry, n (%)	1447 (87.6)	3312 (86.1)	3314 (62.8)	<0.001
male	498 (34.4)	1544 (49.6)	1758 (53.0)	
female	949 (65.6)	1568 (50.4)	1556 (47.0)	
Concentric remodeling, n (%)	106 (6.4)	192 (5.3)	464 (8.8)	<0.001
male	28 (26.4)	70 (36.5)	222 (47.8)	
female	78 (73.6)	122 (63.5)	242 (52.2)	
Concentric hypertrophy, n (%)	13 (0.8)	49 (1.4)	561 (10.6)	<0.001
male	4 (30.8)	21 (42.9)	241 (43.0)	
female	9 (69.2)	28 (57.1)	320 (57.0)	
Eccentric hypertrophy, n (%)	85 (5.1)	263 (7.3)	941 (17.8)	<0.001
male	14 (16.5)	93 (35.4)	332 (35.3)	
female	71 (83.5)	170 (64.6)	609 (64.7)	

### Association of BP indices with left heart remodeling in prehypertension and hypertension group

After adjustment for age, gender, race, height, weight, WC, heart rate, smoking, drinking, salt, degree of physical labor and education, TG, TC, HDL-C, LDL-C, FPG, PC, hemoglobin and UA and antihypertensive medication, logistic regression models were performed to estimate the relationship of four BP indices with LV remodeling in prehypertension and hypertension groups. Results showed that SBP, DBP and MAP were all independent risk factors for left cardiac structural changes, and PP was independent risk factor for concentric and eccentric hypertrophy. Among four BP indices, DBP was the strongest indicator for LV remodeling (Table 4).

**Table 4:** Multiple logistic regression analysis of BP for left heart remodeling in prehypertension and hypertension goups

Classification	Concentric remodeling		Concentric hypertrophy		Eccentric hypertrophy	
	OR (95 % CI)	*P*	OR (95 % CI)	*P*	OR (95 % CI)	*P*
SBP,(+10 mmHg)	1.065 (1.022–1.110)	0.003	1.358 (1.304–1.414)	<0.001	1.159 (1.112–1.198)	<0.001
DBP,(+10 mmHg)	1.192 (1.102–1.290)	<0.001	1.759 (1.623–1.908)	<0.001	1.278 (1.197–1.363)	<0.001
MAP,(+10 mmHg)	1.148 (1.075–1.226)	<0.001	1.700 (1.591–1.817)	<0.001	1.277 (1.211–1.348)	<0.001
PP, (+10 mmHg)	1.025 (0.971–1.082)	0.364	1.314 (1.247–1.384)	<0.001	1.149 (1.101–1.200)	<0.001

Adjustment for age, gender, race, height, weight, WC, heart rate, smoking, drinking, salt, degree of physical labor and education, TG, TC, HDL-C, LDL-C, FPG, PC, hemoglobin, UA and antihypertensive medication

## Discussion

Our research showed that LV remodeling was found in prehypertension group. Although there were no significantly differences in IVSd, PWTd, RWT between prehypertension and normotension group, the values of LVIDd, LVIDs, LVMI, LVEDV, LVESV and SV that reflect ventricular volume indexes were significantly higher in prehypertension group than normotension group, which suggested that there existed cardiac enlargement, LV remodeling and increased LV volume in prehypertensive status. However, hypertension can produce more obvious myocardial cell compensatory enlargement, LV hypertrophy, LV remodeling and volume increases [19]. The prevalence of LVH was statistically different among three groups and the rates of LVH were higher when LVM was indexed to height^2.7^ than to BSA in our study. BSA has been widely used to normalize LVM, however, this does not correctly identify pathological LV hypertrophy in all people, especially when body composition is altered, or in different ethnic groups and may not account for the resultant variation in LVM. Indexation by height^2.7^ may be considered a more suitable approach to scaling LVM for body size in recent years, especially in obese and overweight hypertensive patients, to some extent independently from the cut-off settled. Considering the presence of a significative difference in BMI between the three groups in our study, we indexed LVM by height^2.7^ to classify LV geometry in order to minimize the interference of obesity [19–21]. Impaired LV diastolic dysfunction happened earlier than systolic dysfunction and was followed by a decrease in early-diastolic ventricular filling and a greater atrial contribution to ventricular filling in the late-systolic period [22]. Our study showed that hypertension and prehypertension groups had lower E wave and E/A, but higher A wave. No significant differences in contractility indexes between normotension and prehypertension groups indicated that myocardial total systolic function had not yet been compromised despite LV geometric abnormalities at the stage of prehypertension. LAD, as evidence of diastolic dysfunction, was significantly progressive enlargement from prehypertension to hypertension group.

According to LV hemodynamic changes, LV geometrical change can be classified into three types: concentric remodeling, concentric hypertrophy and eccentric hypertrophy. This is determined to a large extent by whether pressure or volume overload is predominating. Approximately 7.3 % of prehypertensive participants and 17.8 % of hypertensive ones in our study had eccentric hypertrophy which was the highest proportion of abnormal geometrical changes in the our study population. On the one hand, the high prevalence of eccentric hypertrophy may be related to the status of volume overload which play a key role in LV adaptations in hypertension or the high prevalence of obesity [23–25]. On the other hand, subjects in hypertension and prehypertension groups with eccentric geometry are more likely to have coronary artery disease than patients with concentric geometry [26]. Previous studies had indicated that coronary flow reserve was obviously impaired in patients with prehypertension and hypertension, and may occur in the early stage before apparent hypertrophy, which may cause subsequent ischemia, as a consequence of postischemic remodeling [27]. Consistent with our results, some other studies had also showed the similar results that eccentric hypertrophy was the most frequent pathological pattern of LV remodeling [28–30]. However, some studies also reported that concentric remodeling and concentric hypertrophy were the predominant among their studied population with hypertension [31–34]. The difference can partly be explained by difference in echocardiographic cut-off values for defining LVH and the heterogeneity of different population [8]. Besides, our study showed that participants with LV geometrical changes were more likely to be females. It suggested that the women’s hearts were more susceptible to trophic stimuli and neurohumoral factors which may be associated with LV gender-dependent response effect [35, 36]. Eccentric and concentric remodeling were associated with increased weight, BMI, and WC. So in our study, some participants with normotension had LV remodeling. However, LV remodeling was as expected more frequent in the persons suffering from prehypertension or hypertension.

Results from multivariate logistic regression analysis showed that SBP, DBP, MAP and PP were all positively associated with left cardiac structural changes. DBP was the strongest risk factor for concentric remodeling, concentric hypertrophy and eccentric hypertrophy. Thus, it reminds us to strengthen the control of hypertension and pay more attention to prehypertensive status, in order to delay the cardiac damage and the onset of heart failure [37].

This study has some limitations. First, our data is a referral and not a general adult population throughout China. Second, although we made extensive efforts to standardize measurements using the same echocardiographic criteria, subtle differences in performance technique among doctors could still influenced results. In addition, our results were based on a cross-sectional design, thus we are unable to assess the duration of hypertension and antihypertensive medication exactly.

## Conclusions

In this study, there existed LV remodeling and geometrical change in prehypertensive status, and more obvious myocardial cell compensatory enlargement, LV hypertrophy, LV remodeling and volume increases in adults with hypertension in rural Chinese population. The prevalence of LVH was statistically different among three groups and the rates of LVH were higher when LVM was indexed to height^2.7^ than to BSA. The eccentric hypertrophy was the commonest abnormal geometrical pattern in our study and LV geometrical changes were more likely to be females. SBP, DBP, MAP and PP were all positively associated with left cardiac structural changes, and the association of DBP was the strongest.

## Abbreviations

A, atrial peak flow; AOD, Aortic annular diameter; BMI, Body mass index; BP, Blood pressure; BSA, Body surface area; CVD, Cardiovascular disease; DBP, Diastolic blood pressure; E, Early transmitral diastolic peak flow; FPG, Fasting plasma glucose; HDL-C, High-density lipoprotein cholesterol; IVSd, Interventricular septal thickness; LAD, Left atrial diameter; LDL-C, Low-density lipoprotein cholesterol; LV, Left ventricular; LVEDV, LV end-diastolic volume; LVESV, LV end-systolic volume; LVH, Left ventricular hypertrophy; LVIDd, LV end-diastolic internal dimensionn LVIDs; LV end-systolic internal dimension; LVM, Left ventricular mass; LVMI, Left ventricular mass index; MAP, Mean artery pressure; PC, Plasma creatine; PP, Pulse pressure; PWTd, Posterior wall thickness; RWT, Relative wall thickness; SBP, Systolic blood pressure; TC, Total cholesterol; TG, Triglyceride; WC, Waist circumference.
